# Supplementary material for: Widespread inter‐individual gene expression variability in Arabidopsis thaliana
Source: Mol Syst Biol. 2019 Jan 24;15(1):e8591. doi: 10.15252/msb.20188591 (PMC6346214; doi:10.15252/msb.20188591)
Supplement: Supplementary file 1 — Appendix [file MSB-15-e8591-s001.pdf]

Widespread inter-individual gene expression variability in *Arabidopsis thaliana*

Sandra Cortijo[1], Zeynep Aydin[1], Sebastian Ahnert[1], James C.W. Locke[1#]
[1]The Sainsbury Laboratory, University of Cambridge, Cambridge, CB2 1LR, UK
Corresponding author: James Locke, The Sainsbury Laboratory, 47 Bateman St., Cambridge CB2 1LR, UK.
Contact. Tel: +44(0)1223 761110. Email: James.Locke@slcu.cam.ac.uk

Appendix

Table of content

Appendix Figure S1 .... p1
Appendix Figure S2 .... p4
Appendix Figure S3 .... p9
Appendix Figure S4 .... p12
Appendix Figure S5 .... p15
Appendix Figure S6 .... p18
Appendix Figure S7 .... p22

Appendix figure S1

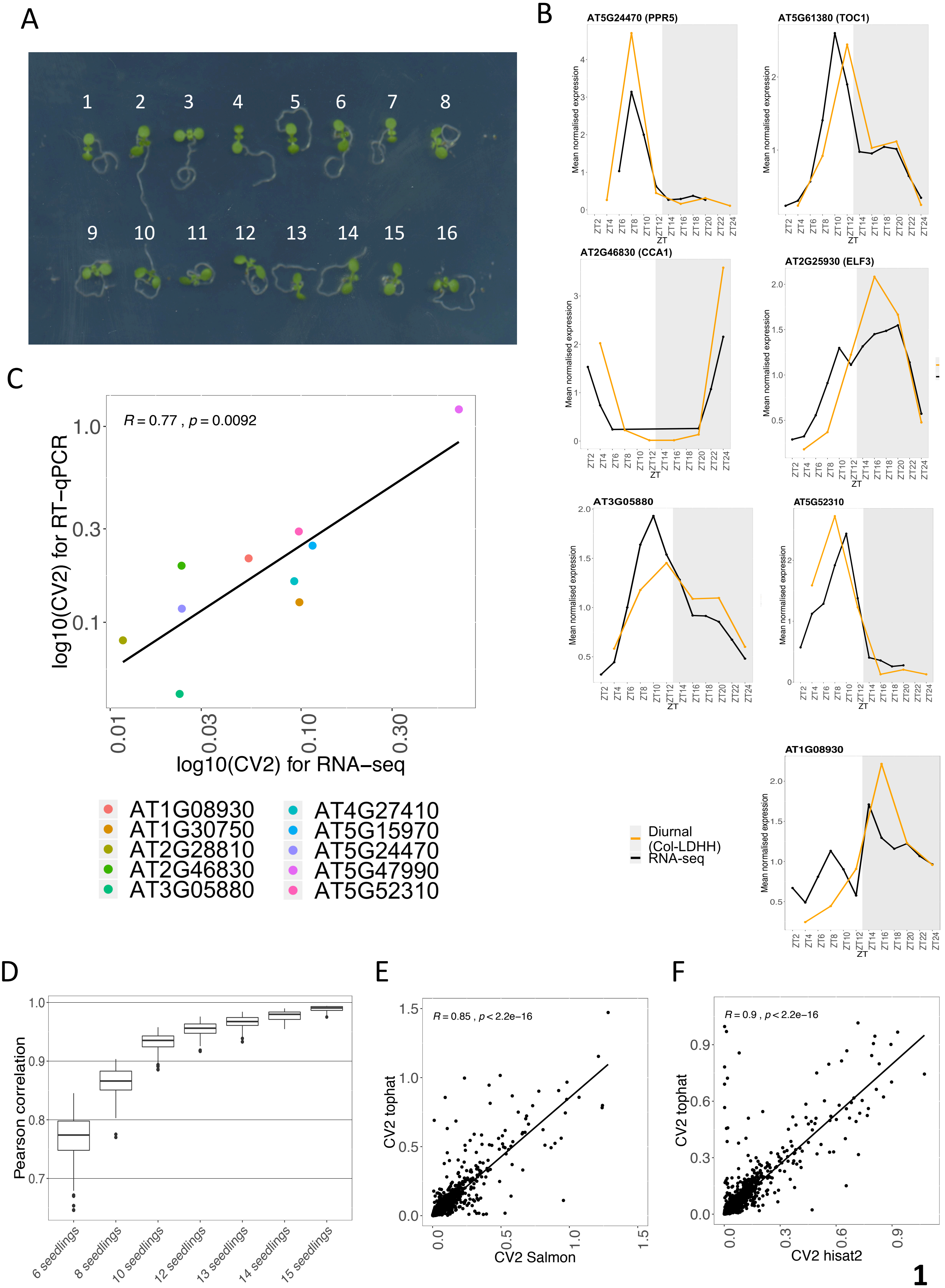

Appendix figure S1 (continued)

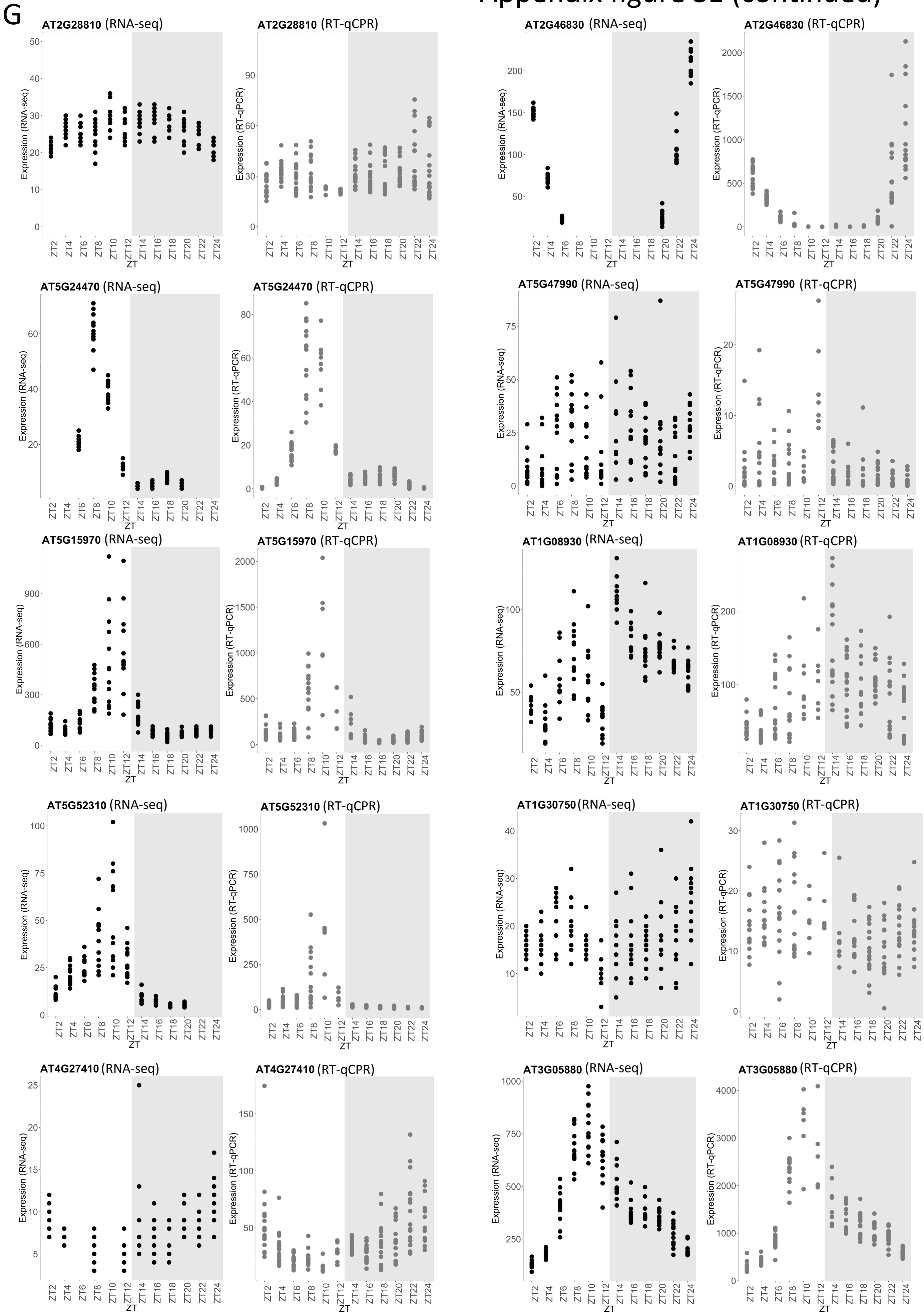

Appendix Figure S1:
A. Photo of seedlings grown in exactly the same conditions used in the project, at the time they would be harvested. Seedling number is indicated in white.
B. Comparison of the average expression profiles (of the 14 seedlings) over the 24 hour time course (with 12 time-points) from our RNA-seq dataset and an already available diurnal expression dataset (http://diurnal.mocklerlab.org). Data from the condition Col-LDHH from the diurnal dataset was used, as its growing conditions are the closest to ours. Expression data for the first 24 hour were used; with a total of 6 time-points (every 4 hours). Each dot is the mean normalised expression level for the available diurnal expression data (orange) or for the average over all 14 seedlings from the RNAseq dataset (black dot).
C. Comparison of the CV^2 measured for 10 genes over the 24 hour time course (with 12 time-points) from the RNA-seq dataset and a replicate experiment carried out by RT-qPCR. Each dot is the average CV^2 over the time course for one of the 10 genes. The Spearman correlation between the CV^2 measured in the RNA-seq and in the RT-qPCR experiments is 0.77, with a p-value of 0.0092.
D. Comparison of the corrected CV^2 calculated from a subset of the data, from 6 to 15 seedlings, with the corrected CV^2 measured from 16 seedlings at ZT6.
E. Comparison of the CV^2 of all genes at ZT24 when mapping is performed with tophat or with salmon (Spearman correlation of 0.85, p-value < 2.2e-16).
F. Comparison of the CV^2 of all genes at ZT24 when mapping is performed with tophat or with hisat2 (Spearman correlation of 0.9, p-value < 2.2e-16).
G. Comparison of the expression profiles in the 14 seedlings over a 24 hour time course (with 12 time-points) in the RNA-seq and in a replicate experiment done by RT-qPCR for 10 genes with different expression and variability profiles. Each dot is the mean normalised expression level for a single seedling from the RNAseq (left plot) or from the RT-qPCR (right plot).

Appendix figure S2

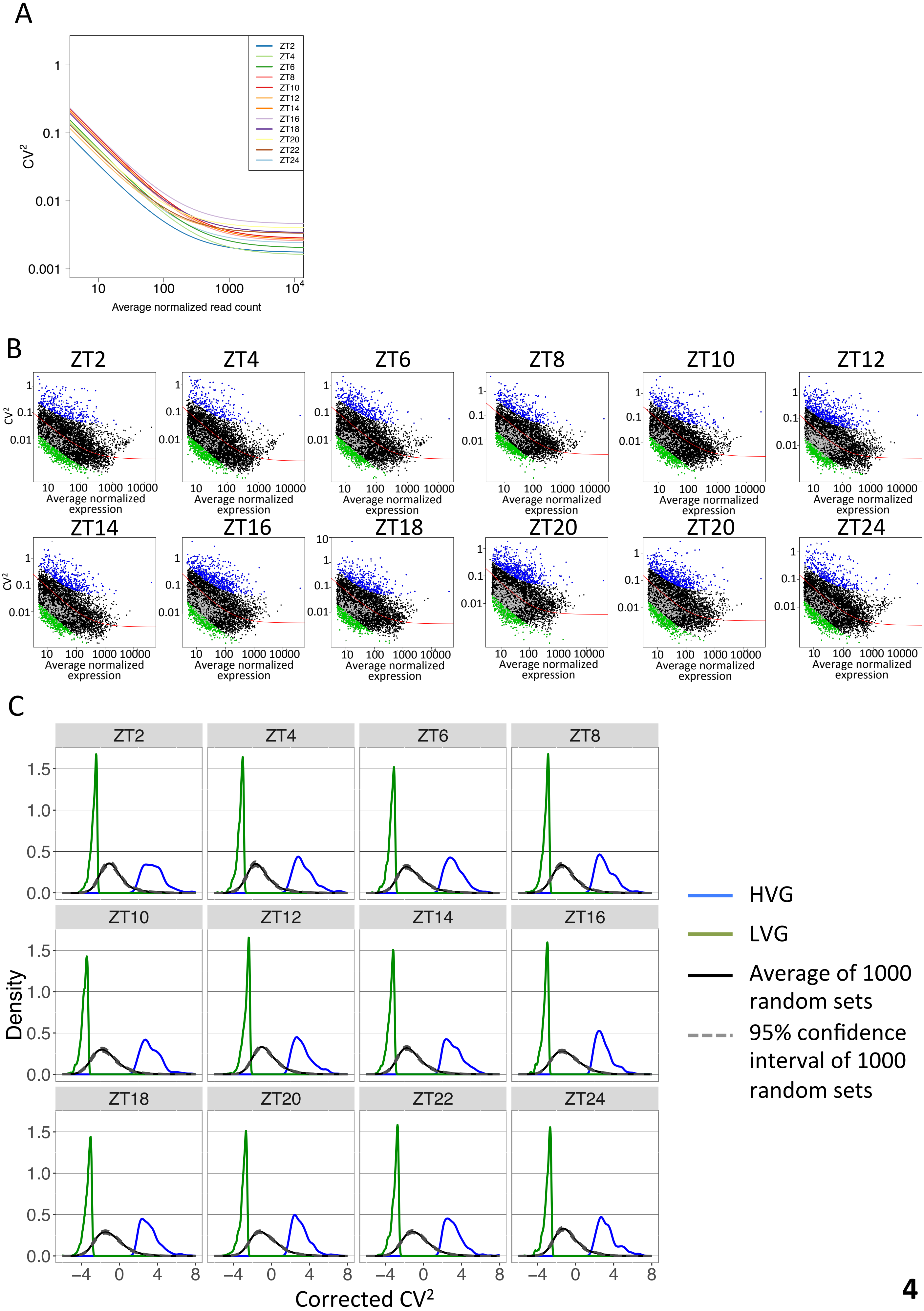

Appendix figure S2 (continued)

D

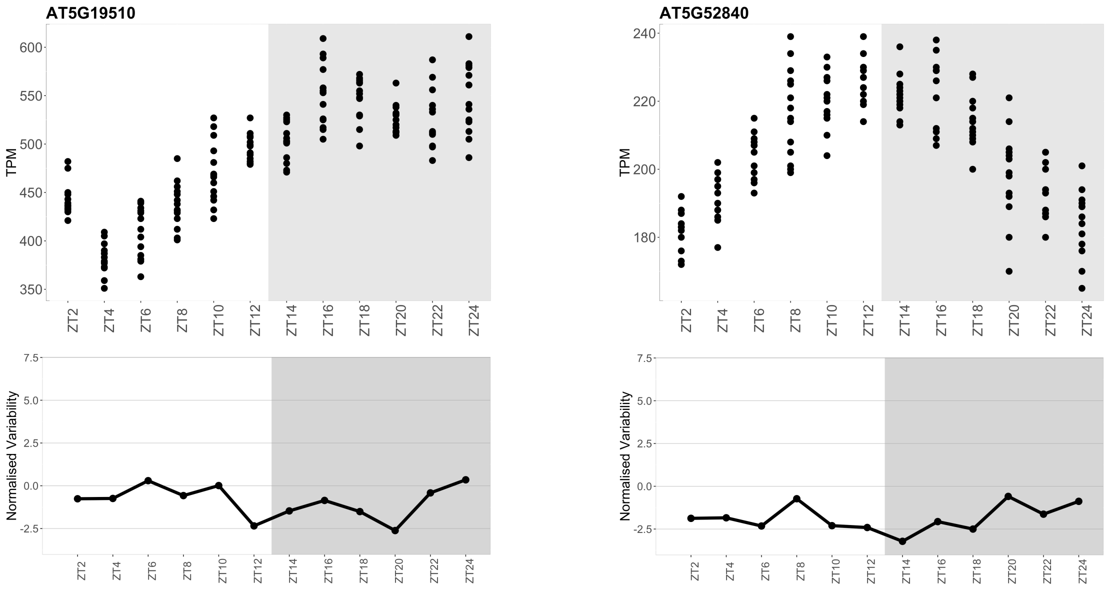

E

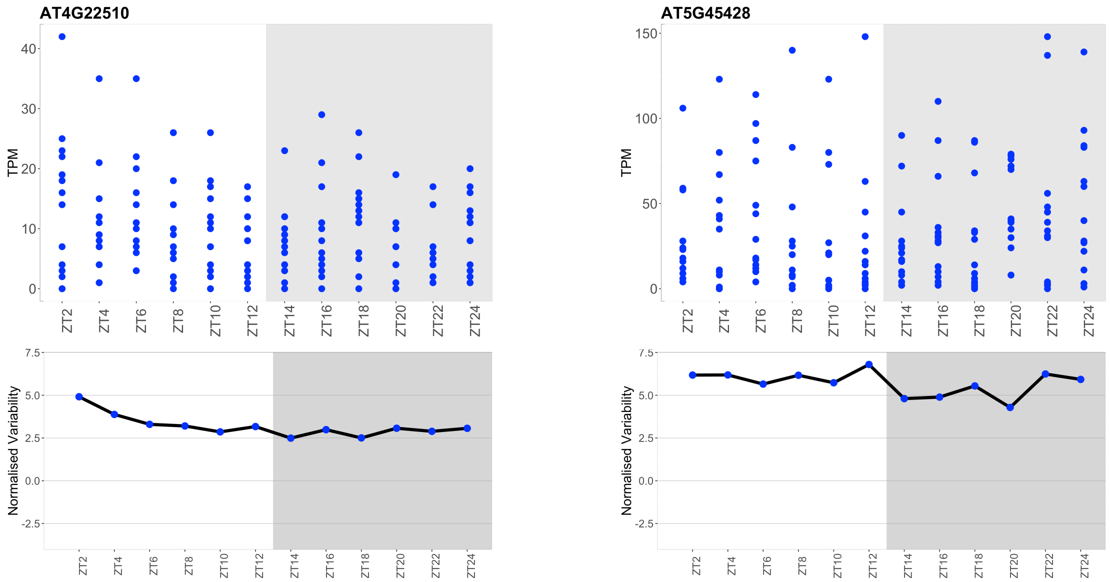

F

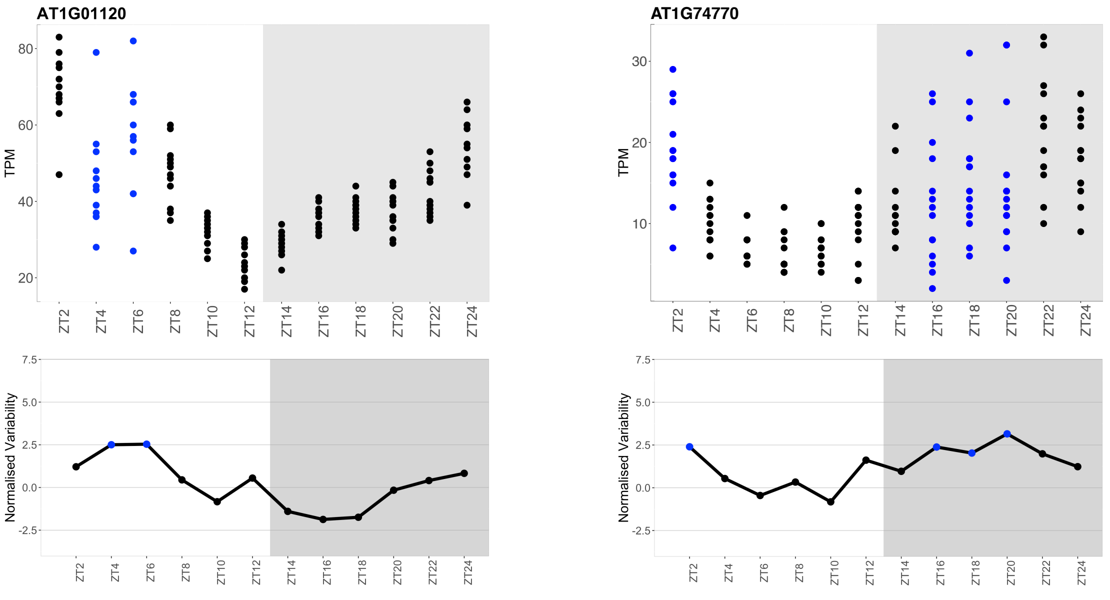

Appendix figure S2 (continued)

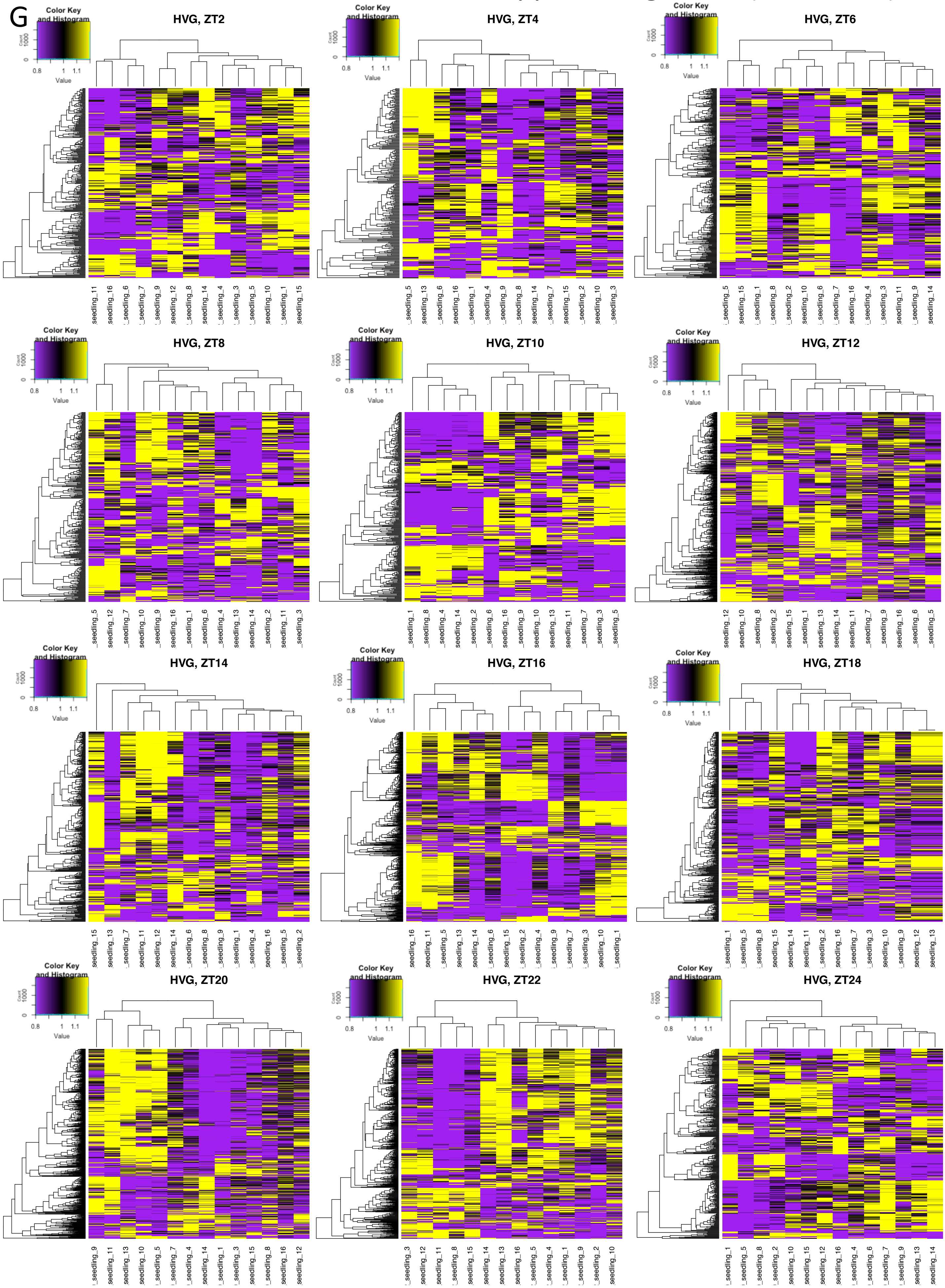

H

About page of AraNoisy

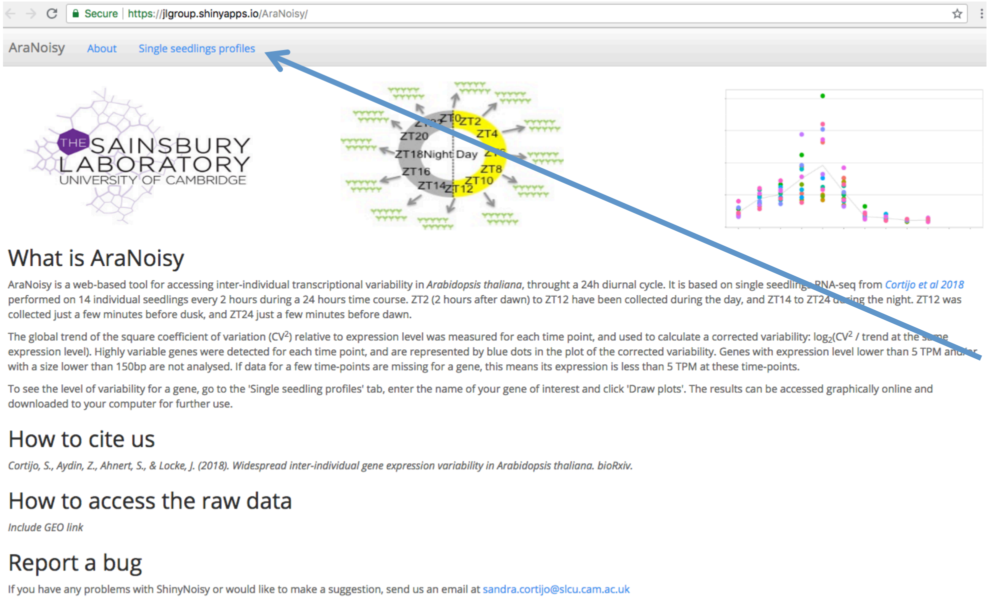

1. To go to AraNoisy tool, click ‘Single seedling profiles’

AraNoisy tool page

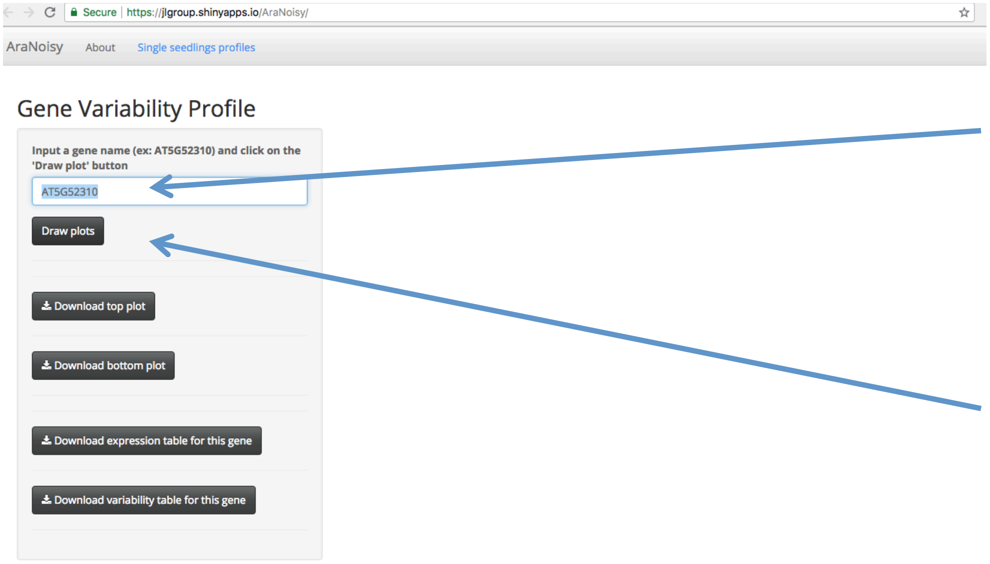

2. Enter a gene id, using the following format: AT5G52310

3. Click ‘Draw plot’
You need to click ‘Draw plot’ each time you enter a new gene id

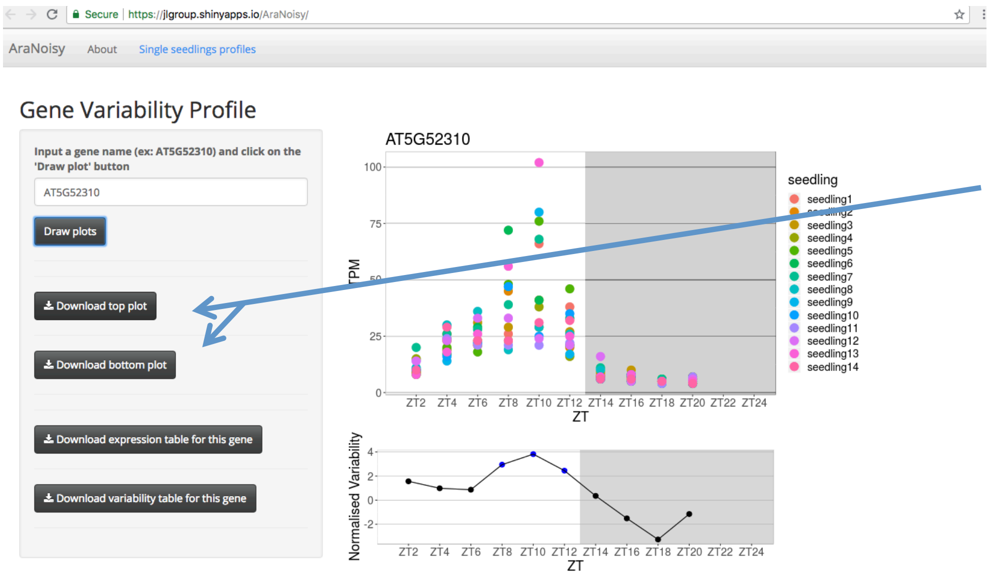

4. Click to download the top plot (expression levels during the time course in the single seedlings) or the bottom plot (variability level during the time course)

Appendix Figure S2:

A. Trend for the global CV^2 profile for each of the 12 time-points.

B. Identification of HVGs (blue), LVGs (green) and of one set of random genes (grey) for each of the 12 time-points.

C. Distribution of the corrected CV^2 for LVGs (green) HVGs (blue) at each time-point. The distribution of average (black) and 95% confidence interval (dotted grey) for the thousand sets of random genes (of same size as HVGs) is also represented at each time-point.

D. Expression profiles (top) in the 14 seedlings over a 24 hour time course (with 12 time-points) for two non-variable genes. Each dot is expression level (TPM) for a single seedling. Variability profiles (bottom) of the $\log_2(CV^2/\text{trend})$ for the same genes are also shown.

E. Expression profiles (top) in the 14 seedlings over a 24 hour time course (with 12 time-points) for two highly variable genes. Each dot is the expression level (TPM) for a single seedling. Variability profiles (bottom) of the $\log_2(CV^2/\text{trend})$ for the same genes are also shown. Blue dots indicate time-points for which the gene is identified as being variable.

F. Expression profiles (top) in the 14 seedlings over a 24 hour time course (with 12 time-points) for two genes with the level of variability changing across the 24 hour. Each dot is the expression level (TPM) for a single seedling. Variability profiles (bottom) of the $\log_2(CV^2/\text{trend})$ for the same genes are also shown. Blue dots indicate time-points for which the gene is identified as being variable.

G. Hierarchical clustering for each time-point of HVGs and of individual seedling using the mean normalised expression levels. The result is represented as a heatmap where yellow indicates a high mean normalised expression.

H. Step by step guide showing how to use the AraNoisy web-application (https://jlgroup.shinyapps.io/AraNoisy/). The top panel shows the about page, which contains a description of the tool. The page with the AraNoisy tool is accessed by clicking on the 'Single seedlings profiles' tab. This page and explanation of how to generate graphs, and export them, for variability profiles and expression profiles in the 14 seedlings are detailed in the middle and bottom panels.

Appendix figure S3

A

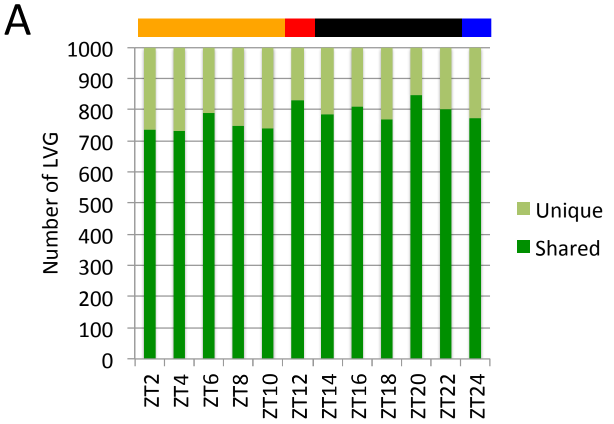

B

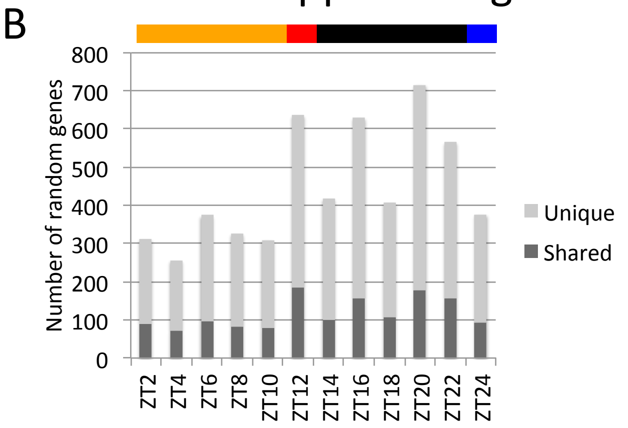

C

Percentage of genes shared, LVG

	ZT2	ZT4	ZT6	ZT8	ZT10	ZT12	ZT14	ZT16	ZT18	ZT20	ZT22	ZT24
ZT2	/	14.4	17.7	13.4	16.2	16.7	15.2	19.3	17.2	18.8	20.4	18
ZT4	14.4	/	18.5	16	13.1	19.8	21.1	14	17.5	15.3	17.4	14.8
ZT6	17.7	18.5	/	19	20.7	22.9	19.2	21.1	17.6	20	17.8	17.3
ZT8	13.4	16	19	/	25.3	21.6	17.4	16.4	11.5	16.9	10.7	15.8
ZT10	16.2	13.1	20.7	25.3	/	22.2	15.7	16.5	13.3	16	12.1	14.8
ZT12	16.7	19.8	22.9	21.6	22.2	/	20.1	19.3	18.6	20.3	17.6	18.1
ZT14	15.2	21.1	19.2	17.4	15.7	20.1	/	20.4	18.8	21.3	18	18.3
ZT16	19.3	14	21.1	16.4	16.5	19.3	20.4	/	16.6	26.7	20.9	22.2
ZT18	17.2	17.5	17.6	11.5	13.3	18.6	18.8	16.6	/	20.1	22.6	18.5
ZT20	18.8	15.3	20	16.9	16	20.3	21.3	26.7	20.1	/	24.1	23
ZT22	20.4	17.4	17.8	10.7	12.1	17.6	18	20.9	22.6	24.1	/	19.9
ZT24	18	14.8	17.3	15.8	14.8	18.1	18.3	22.2	18.5	23	19.9	/

D

Percentage of genes shared, random genes

	ZT2	ZT4	ZT6	ZT8	ZT10	ZT12	ZT14	ZT16	ZT18	ZT20	ZT22	ZT24
ZT2	/	2.7	1.9	2.1	1.0	2.8	1.9	1.6	2.2	2.2	1.9	1.1
ZT4	2.7	/	1.6	1.2	2.9	1.7	2.2	1.3	1.0	0.6	2.3	1.9
ZT6	1.9	1.6	/	2.8	1.0	3.3	1.9	1.7	3.2	1.8	2.3	2.1
ZT8	2.1	1.2	2.8	/	1.0	1.9	1.9	2.1	1.5	2.1	3.2	2.1
ZT10	1.0	2.9	1.0	1.0	/	1.6	1.7	2.1	1.7	2.5	1.8	2.4
ZT12	2.8	1.7	3.3	1.9	1.6	/	4.8	5.4	5.1	5.2	3.5	4.2
ZT14	1.9	2.2	1.9	1.9	1.7	4.8	/	2.1	1.7	2.0	3.0	2.1
ZT16	1.6	1.3	1.7	2.1	2.1	5.4	2.1	/	2.9	3.8	4.2	2.4
ZT18	2.2	1.0	3.2	1.5	1.7	5.1	1.7	2.9	/	2.8	3.5	2.4
ZT20	2.2	0.6	1.8	2.1	2.5	5.2	2.0	3.8	2.8	/	5.1	3.4
ZT22	1.9	2.3	2.3	3.2	1.8	3.5	3.0	4.2	3.5	5.1	/	4.2
ZT24	1.1	1.9	2.1	2.1	2.4	4.2	2.1	2.4	2.4	3.4	4.2	/

E

$Log_2(CV^2/trend)$, LVG

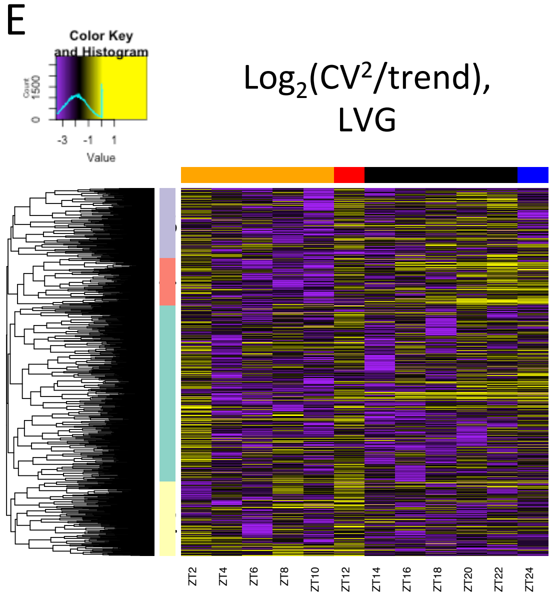

F

$Log_2(CV^2/trend)$, random genes

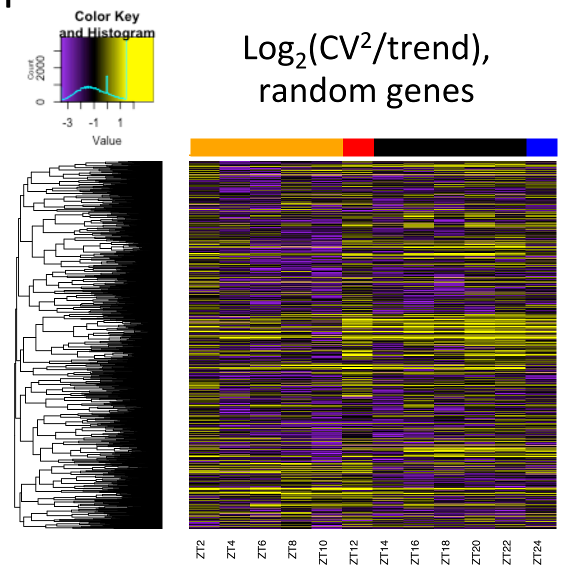

G

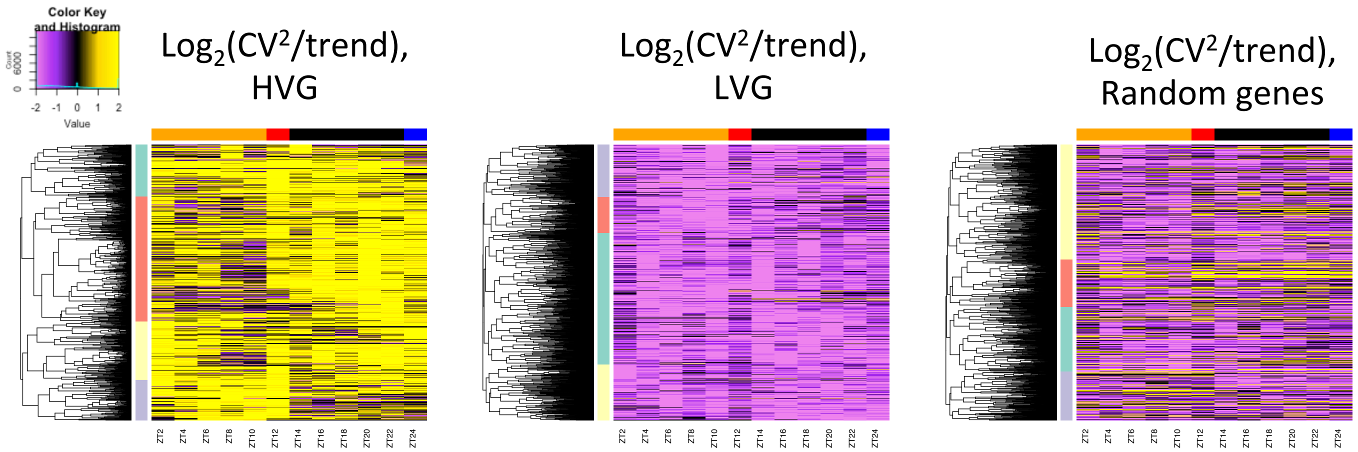
Color Key
and Histogram
Count
Value
Log2(CV2/trend),
HVG
Log2(CV2/trend),
LVG
Log2(CV2/trend),
Random genes
ZT2
ZT4
ZT6
ZT8
ZT10
ZT12
ZT14
ZT16
ZT18
ZT20
ZT22
ZT24

H

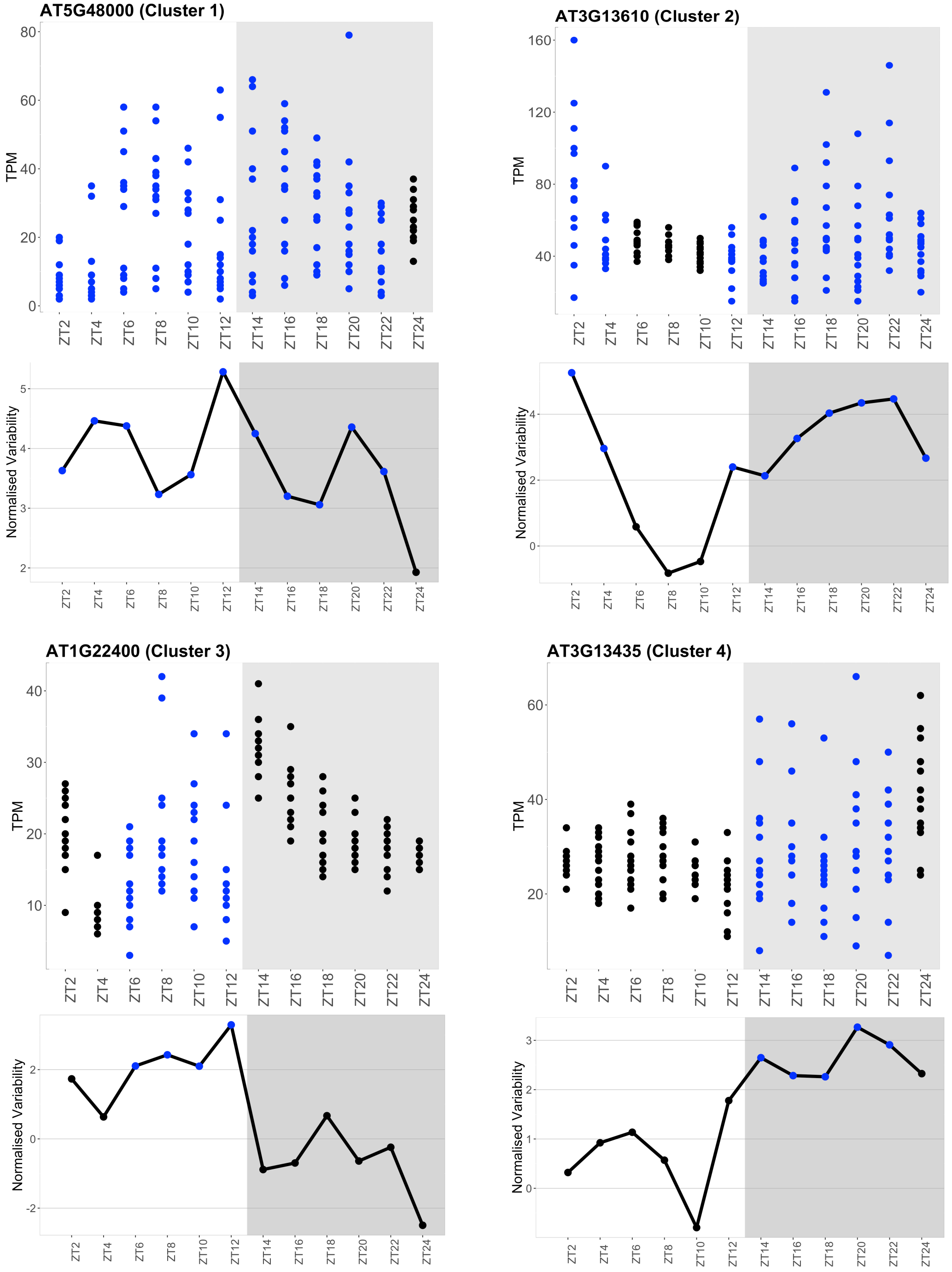
AT5G48000 (Cluster 1)
AT3G13610 (Cluster 2)
AT1G22400 (Cluster 3)
AT3G13435 (Cluster 4)
TPM
Normalised Variability
ZT2
ZT4
ZT6
ZT8
ZT10
ZT12
ZT14
ZT16
ZT18
ZT20
ZT22
ZT24

Appendix Figure S3:

A. Number of LVGs for each time-point. These genes are separated between those that are also LVGs in at least one other time-point ('shared', dark green) or selected in only one time-point ('unique'. light green). The top bar indicates time-points harvested during the day (orange), just before dusk (red), during the night (black) and just before dawn (blue).

B. Example for one set of random genes selected for each time-point, corresponding to the number of HVGs at the corresponding time-point. These genes are separated between those that are also selected in at least one other time-point (dark grey) and those selected in only one time-point (light grey). The top bar indicates time-points harvested during the day (orange), just before dusk (red), during the night (black) and just before dawn (blue).

C. Heatmap of the percentage of LVGs shared between time-points. Red indicates a high percentage in common between two time-points. The top and side bars indicates time-points harvested during the day (orange), just before dusk (red), during the night (black) and just before dawn (blue).

D. Heatmap of the percentage of random genes shared between time-points. Red indicates a high percentage in common between two time-points. The top and side bars indicates time-points harvested during the day (orange), just before dusk (red), during the night (black) and just before dawn (blue).

E. Hierarchical clustering of LVGs based on the $log_2(CV^2/trend)$ at each time point. The result is represented as a heatmap where yellow indicates a high $log_2(CV^2/trend)$. The genes were separated into four clusters, indicated by the side colored bar. The top bar indicates time-points harvested during the day (orange), just before dusk (red), during the might (black) and just before dawn (blue). See Appendix Fig S3G for heatmaps with the same colour cutoffs for HVGs, LVGs and random genes.

F. Hierarchical clustering of one set of random genes based on the $log_2(CV^2/trend)$ at each time point. The result is represented as a heatmap where yellow indicates a high $log_2(CV^2/trend)$. The genes were separated into four clusters, indicated by the side colored bar. The top bar indicates time-points harvested during the day (orange), just before dusk (red), during the night (black) and just before dawn (blue). See Appendix Fig S3G for heatmaps with the same colour cutoffs for HVGs, LVGs and random genes.

G. Hierarchical clustering of HVG (left), LVG (middle) and one set of random genes (right) based on the $log_2(CV^2/trend)$ at each time point. The result is represented as a heatmap where yellow indicates a high $log_2(CV^2/trend)$. The same color cutoff is used in all three heatmaps.

H. Example of expression levels (TPM) in the 14 seedlings across the 12 time-points (top panel) for 4 genes that are representative of each of the 4 clusters identified in Fig 2D for HVGs based on the $log_2(CV^2/trend)$. Each dot is the expression level (TPM) for a single seedling from the RNAseq. AT5G48000, AT3G13610, AT1G22400 and AT3G13435 are representative genes of the clusters 1, 2, 3 and 4 respectively. The normalised CV^2 over the time course (bottom panel) is also shown. Blue dots indicate time-points for which the gene is identified as being highly variable.

Appendix figure S4

A

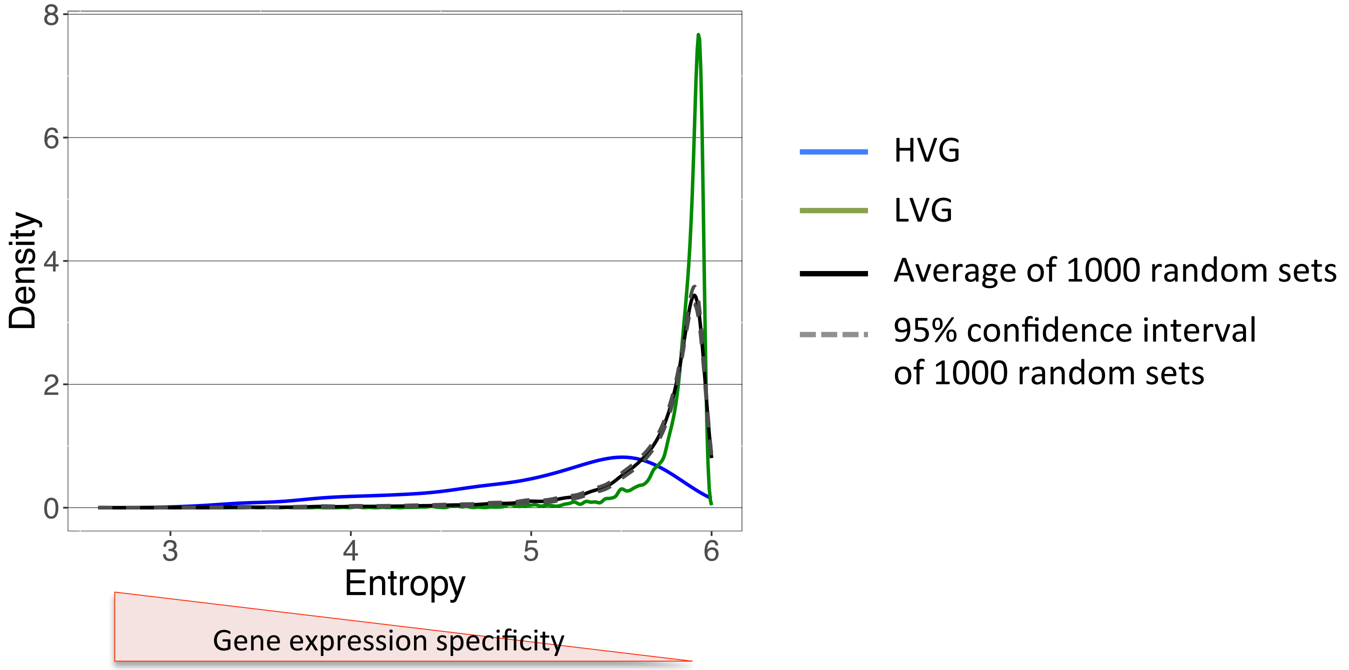

B

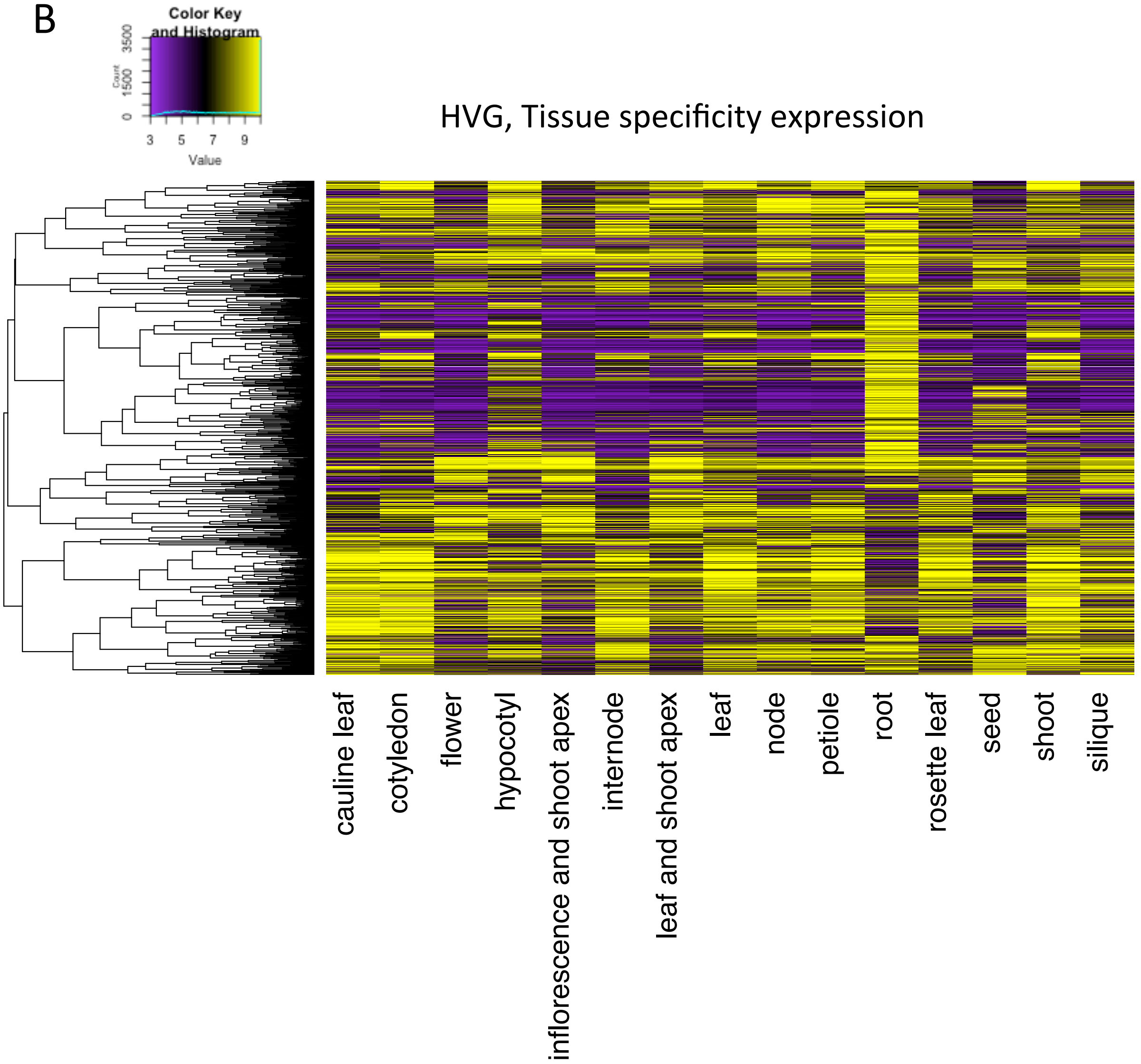

C

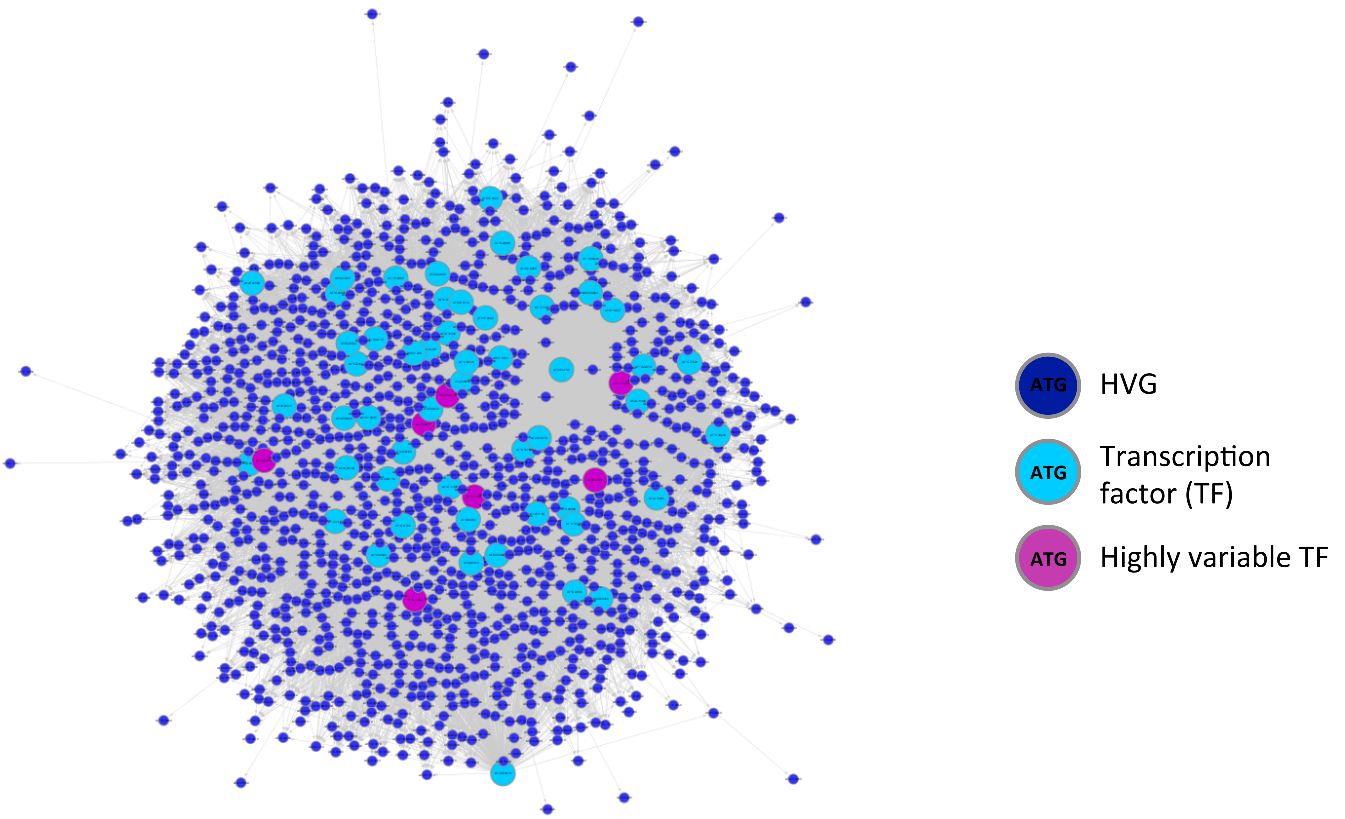
ATG
HVG
ATG
Transcription factor (TF)
ATG
Highly variable TF

D

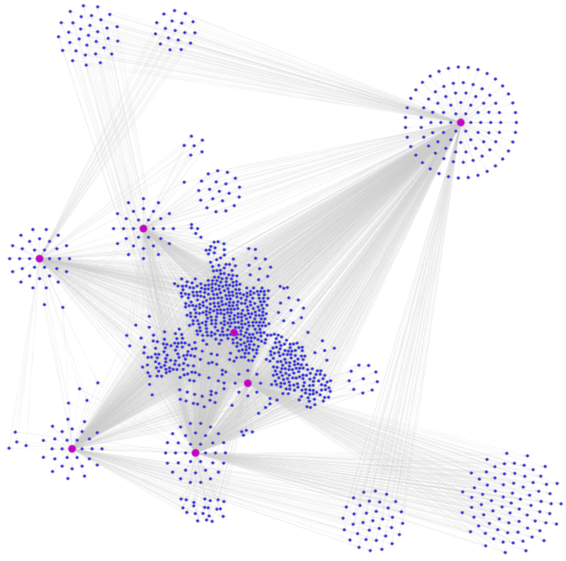

E

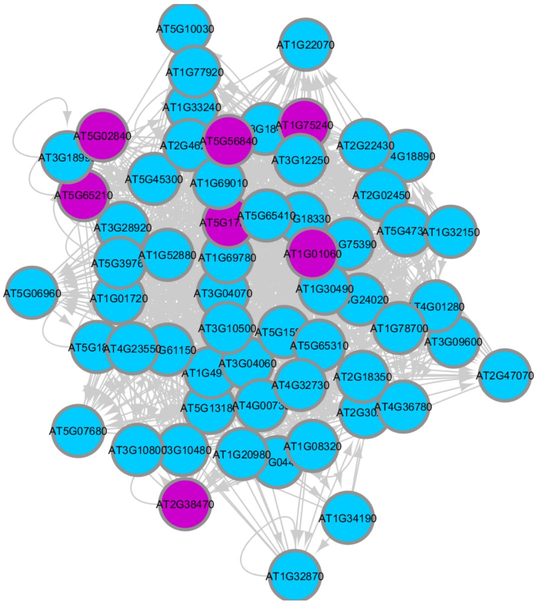
AT5G10030
AT1G22070
AT1G77920
AT1G33240
AT1G75240
AT5G02840
AT5G56840
AT2G22430
AT3G12250
AT5G45300
AT1G69010
AT2G02450
AT5G65210
AT5G65410
AT3G28920
AT1G32150
AT1G52880
AT1G69780
AT1G01060
AT5G06960
AT3G04070
AT1G30490
AT1G01720
AT3G10500
AT1G78700
AT3G09600
AT5G65310
AT3G04060
AT2G18350
AT2G47070
AT4G32730
AT4G36780
AT5G07680
AT1G20980
AT1G08320
AT2G38470
AT1G34190
AT1G32870

Appendix Figure S4:

A. Distribution of the tissue specificity of HVGs (blue) and LVGs (green) as estimated by Shannon entropy calculation. The distribution of average (black) and 95% confidence interval (dotted grey) for the thousand random sets is also represented. Low entropy values indicate high tissue specificity.

B. Hierarchical clustering of HVGs based on the expression level in different tissues. The result is represented as a heatmap where yellow indicates a high expression level.

C. Gene regulatory network derived from the DAP-seq data for HVGs and the 60 TFs with targets enriched in HVGs. Dark blue nodes are HVGs, light blue nodes are TFs and purple nodes are highly variable TFs.

D. Gene regulatory network derived from the DAP-seq data for HVGs and the 7 highly variable TFs with targets enriched in HVGs. Dark blue nodes are HVGs and purple nodes are highly variable TFs.

E. Gene regulatory network derived from the DAP-seq data for 60 TFs with targets enriched in HVGs. Light blue nodes are TFs and purple nodes are highly variable TFs.

Appendix figure S5

A

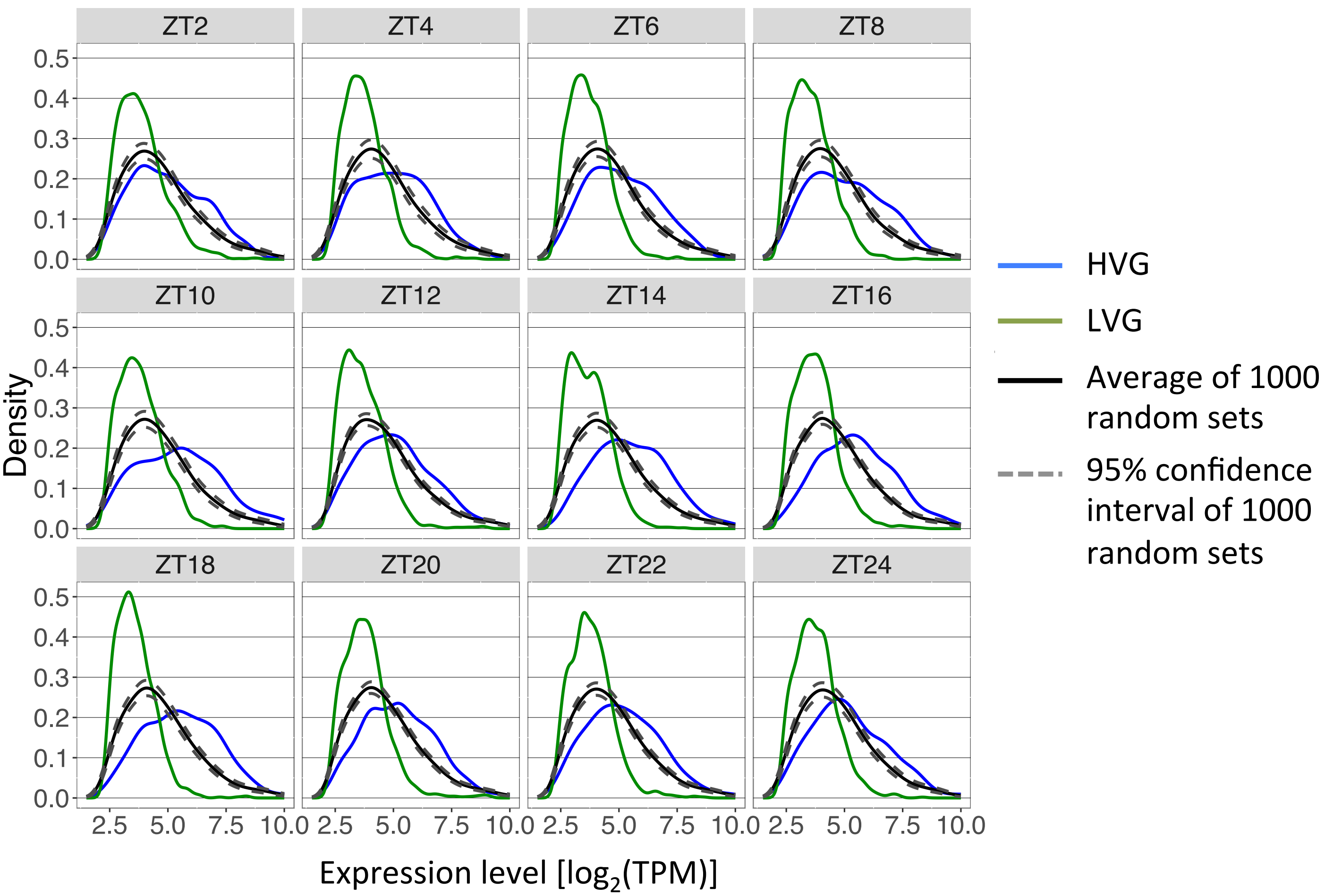

B

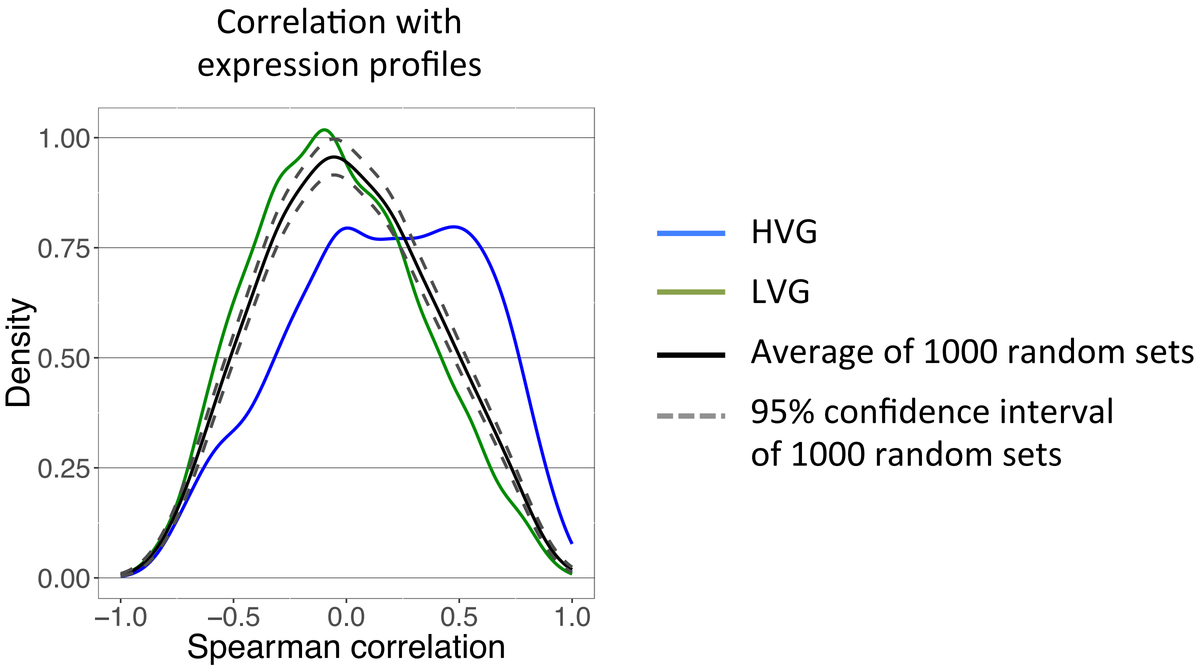

Appendix figure S5 (continued)

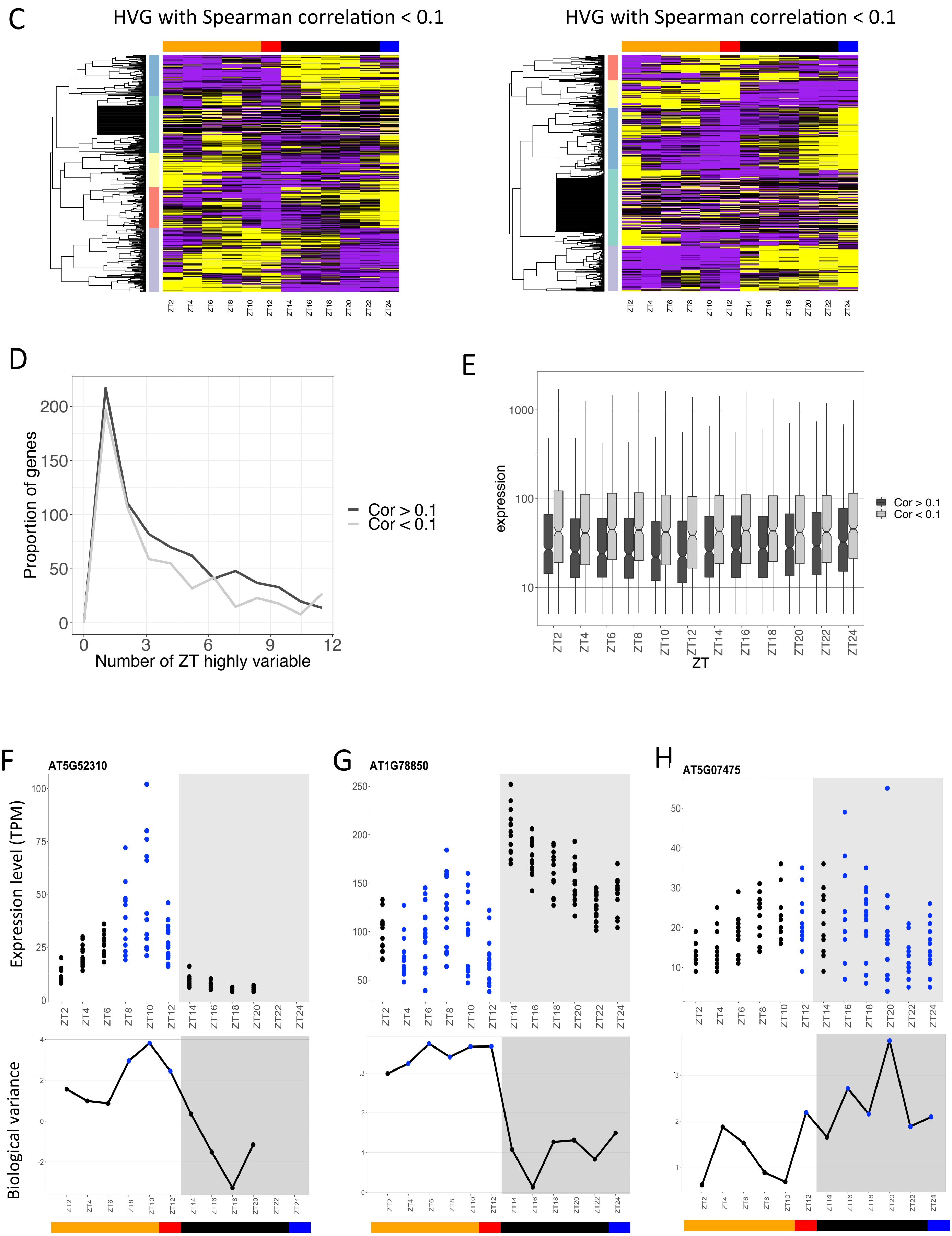

Appendix Figure S5:

A. Distribution of the gene expression levels (log_2(TPM)) for LVGs (green) and HVGs (blue) in each of the 12 time-points. The distribution of average (black) and 95% confidence interval (dotted grey) for the thousand sets of random genes (of same size as HVGs) is also represented at each time-point.

B. Distribution of the Spearman correlation between the average expression profile and the $log_2(CV^2/trend)$ profile for LVG (green) and HVG (blue). The distribution of average (black) and 95% confidence interval (dotted grey) for the thousand random sets is also represented.

C. Hierarchical clustering of HVGs for which the Spearman correlation between the average expression profile and the $log_2(CV^2/trend)$ is less than 0.1 (left) or more than 0.1 (right) based on mean normalised expression level in the 12 time-points. The result is represented as a heatmap where yellow indicates a high mean normalised expression level. The genes were separated into five clusters, indicated by the side colored bar. The top bar indicates time-points harvested during the day (orange), just before dusk (red), during the night (black) and just before dawn (blue).

D. Distribution of the number of time-points at which genes are identified as highly variable, for HVGs for which the Spearman correlation between the average expression profile and the $log_2(CV^2/trend)$ is less than 0.1 (light grey) or more than 0.1 (dark grey).

E. Boxplot of the expression level at each time-point for HVGs for which the Pearson correlation between the average expression profile and the $log_2(CV^2/trend)$ is less than 0.1 (light grey) or more than 0.1 (dark grey).

F. Example of expression levels (TPM) in the 14 seedlings across the 12 time-points (top panel) for a HVG with a strong positive Spearman correlation (0.86) between the average expression profile and the variability profile. The normalised CV^2 over the time course (bottom panel) is also shown. Blue dots indicate time-points for which the gene is identified as being highly variable.

G. Example of expression levels (TPM) in the 14 seedlings across the 12 time-points (top panel) for a HVG with a strong negative Spearman correlation (-0.84) between the average expression profile and the variability profile. The normalised CV^2 over the time course (bottom panel) is also shown. Blue dots indicate time-points for which the gene is identified as being highly variable.

H. Example of expression levels (TPM) in the 14 seedlings across the 12 time-points (top panel) for a HVG without a strong Spearman correlation (0.06) between the average expression profile and the variability profile. The normalised CV^2 over the time course (bottom panel) is also shown. Blue dots indicate time-points for which the gene is identified as being highly variable.

Appendix figure S6

A

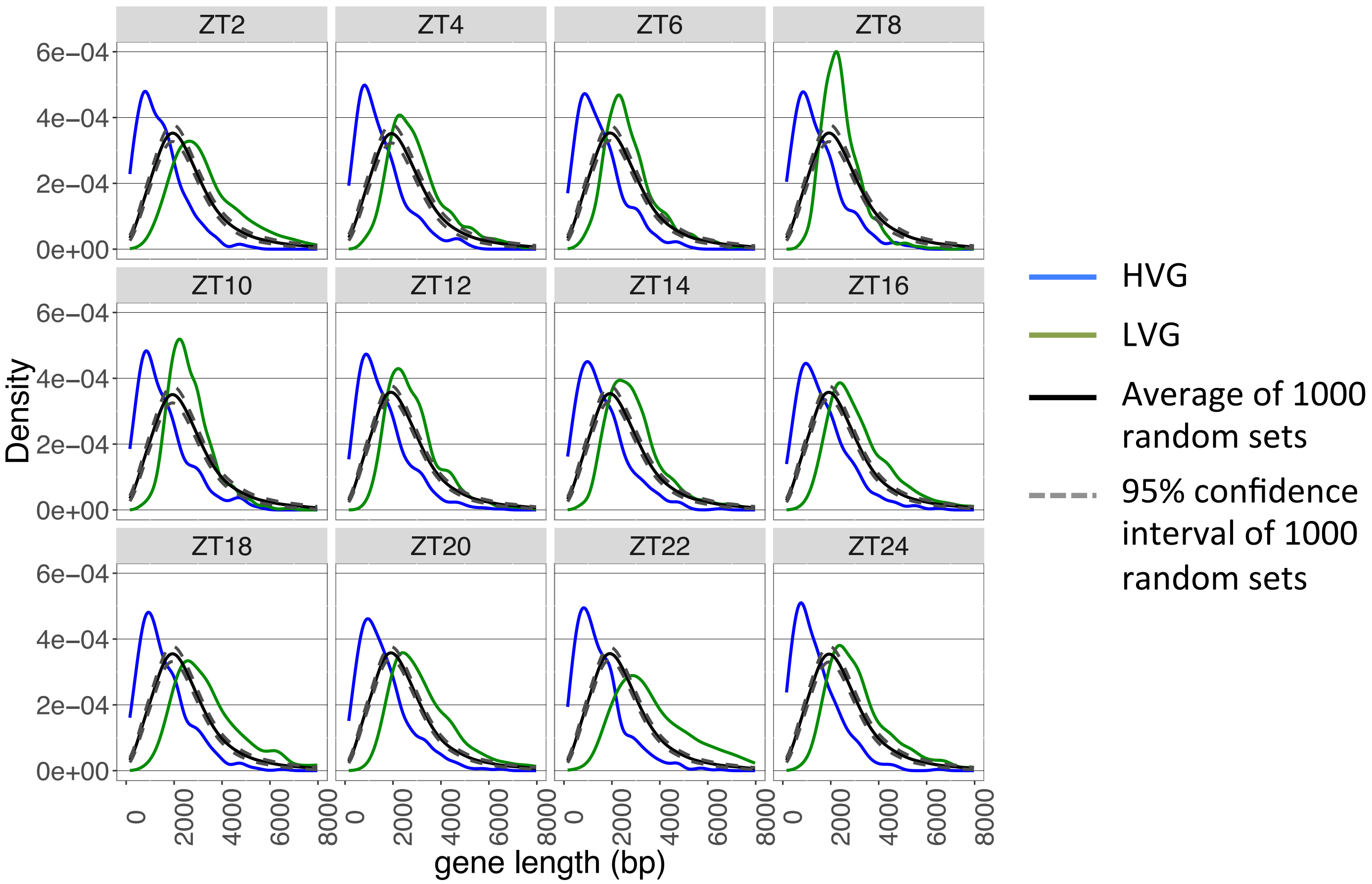

B

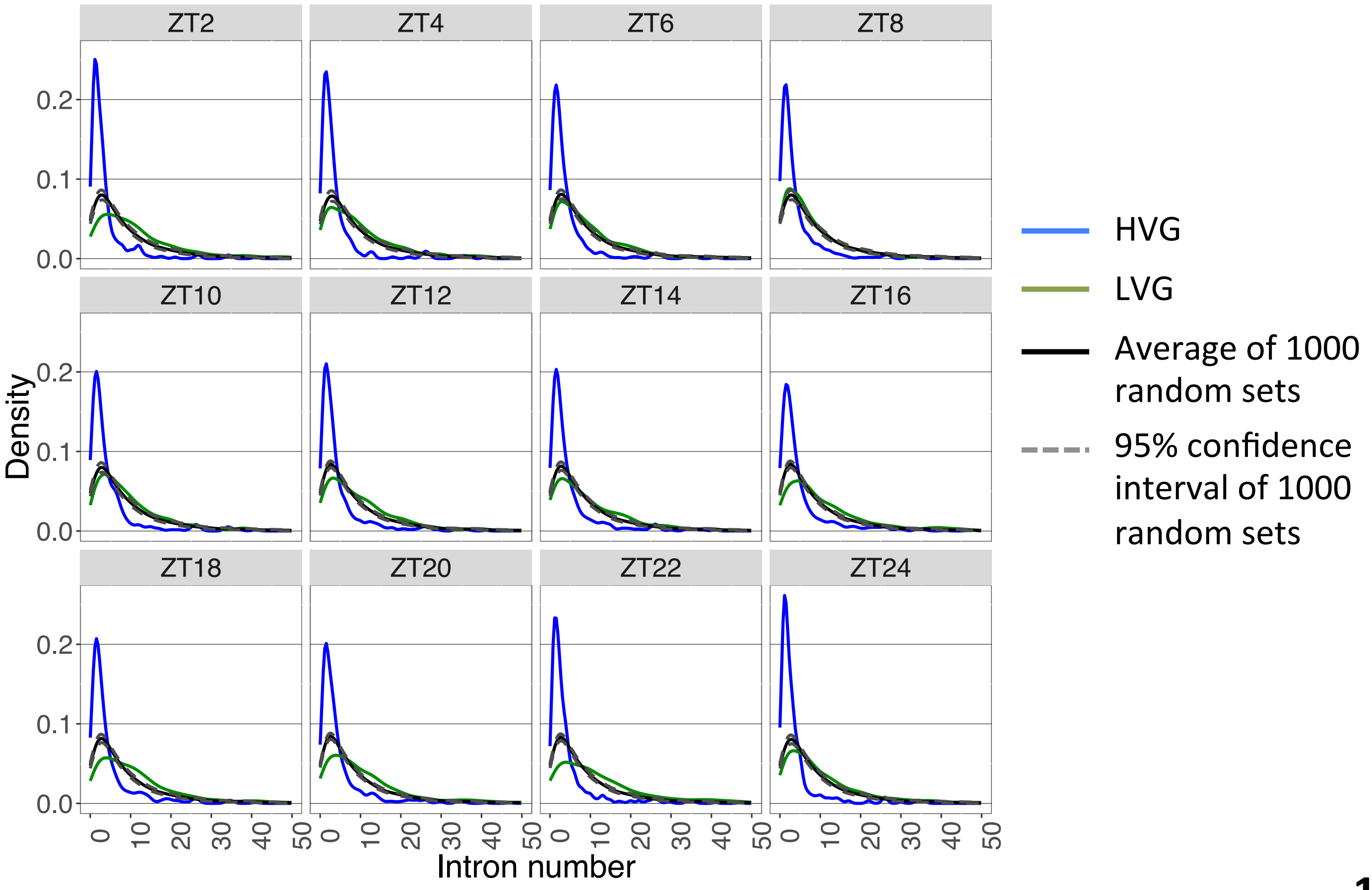

Appendix figure S6 (continued)

C

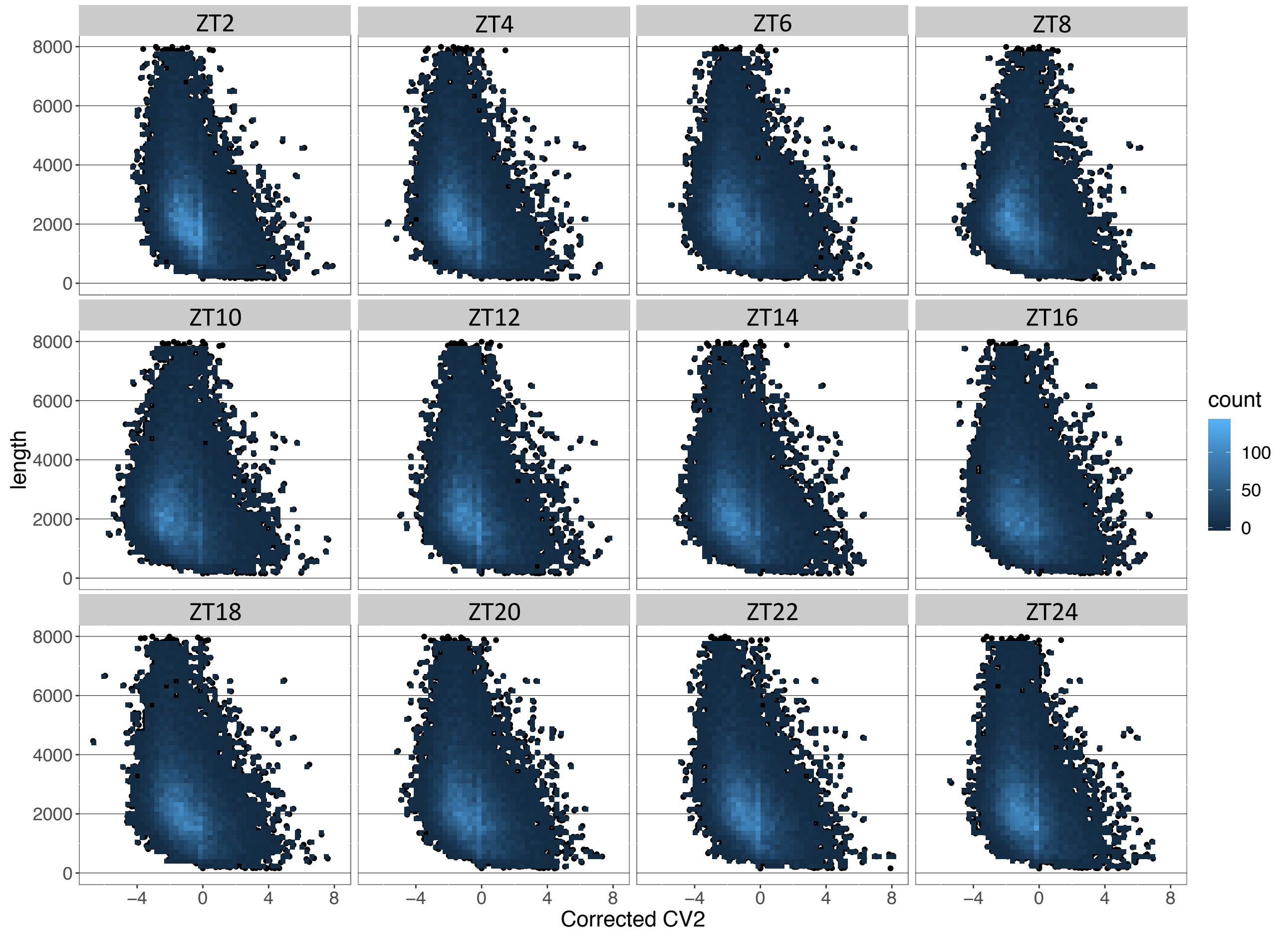

D

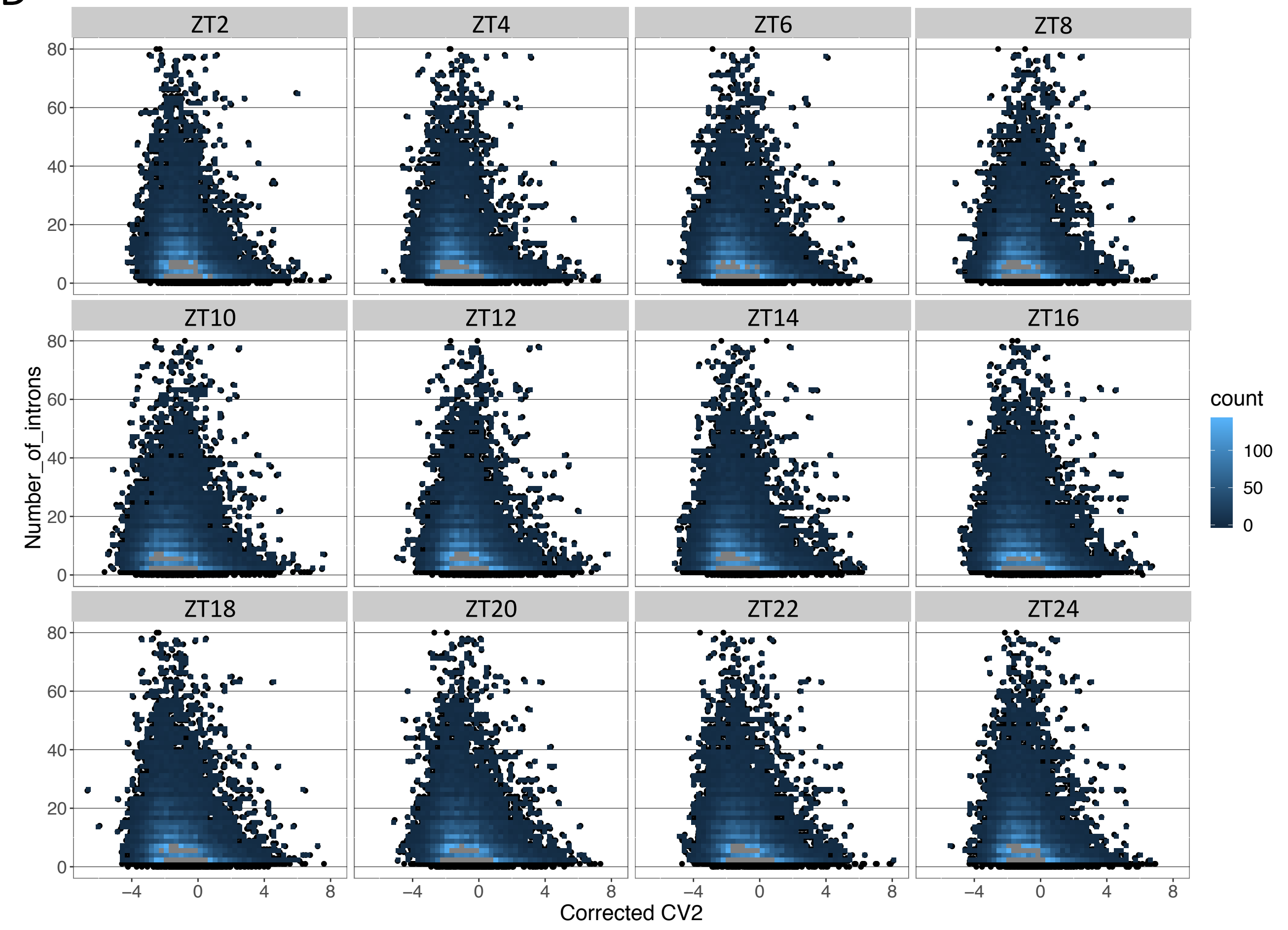

Appendix figure S6 (continued)

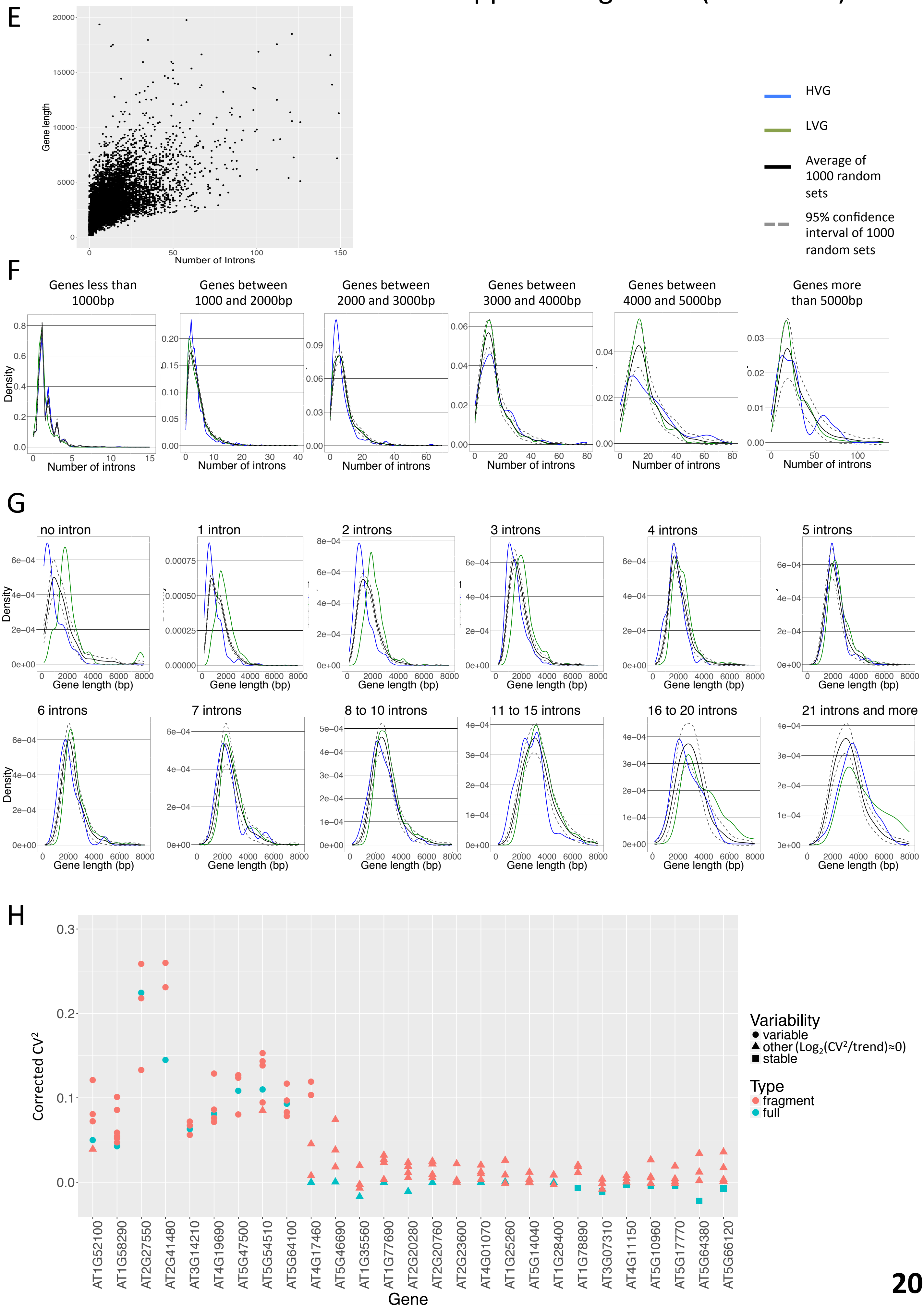

Appendix Figure S6:

A. Distribution of the gene length for LVGs (green) and HVGs (blue) in each time-point. The distribution of average (black) and 95% confidence interval (dotted grey) for the thousand sets of random genes (of same size as HVGs) is also represented at each time-point.

B. Distribution of the number of introns for LVGs (green) and HVGs (blue) in each time-point. The distribution of average (black) and 95% confidence interval (dotted grey) for the thousand sets of random genes (of same size as HVGs) is also represented at each time-point.

C. Plot of the gene length versus the $\log_2(CV^2/\text{trend})$ for all genes in each time-point.

D. Plot of the number of introns versus the $\log_2(CV^2/\text{trend})$ for all genes in each time-point.

E. Plot of the gene length versus the number of introns for all genes.

F. Distribution of the number of introns for LVGs (green) and HVGs (blue) for genes with several windows of gene length. The distribution of average (black) and 95% confidence interval (dotted grey) for the thousand sets of random genes (of same size as HVGs) is also represented at each time-point.

G. Distribution of the gene length for LVGs (green) and HVGs (blue) for genes with fixed number of introns. The distribution of average (black) and 95% confidence interval (dotted grey) for the thousand sets of random genes (of same size as HVGs) is also represented at each time-point.

H. Corrected CV^2 for 9 HVG (round), 7 LVG (square) and 11 other genes (triangle), for the full genes (blue) as well as ~250bp fragments of these genes (red). Variability levels of full genes and their fragments were analysed together.

Appendix figure S7

A

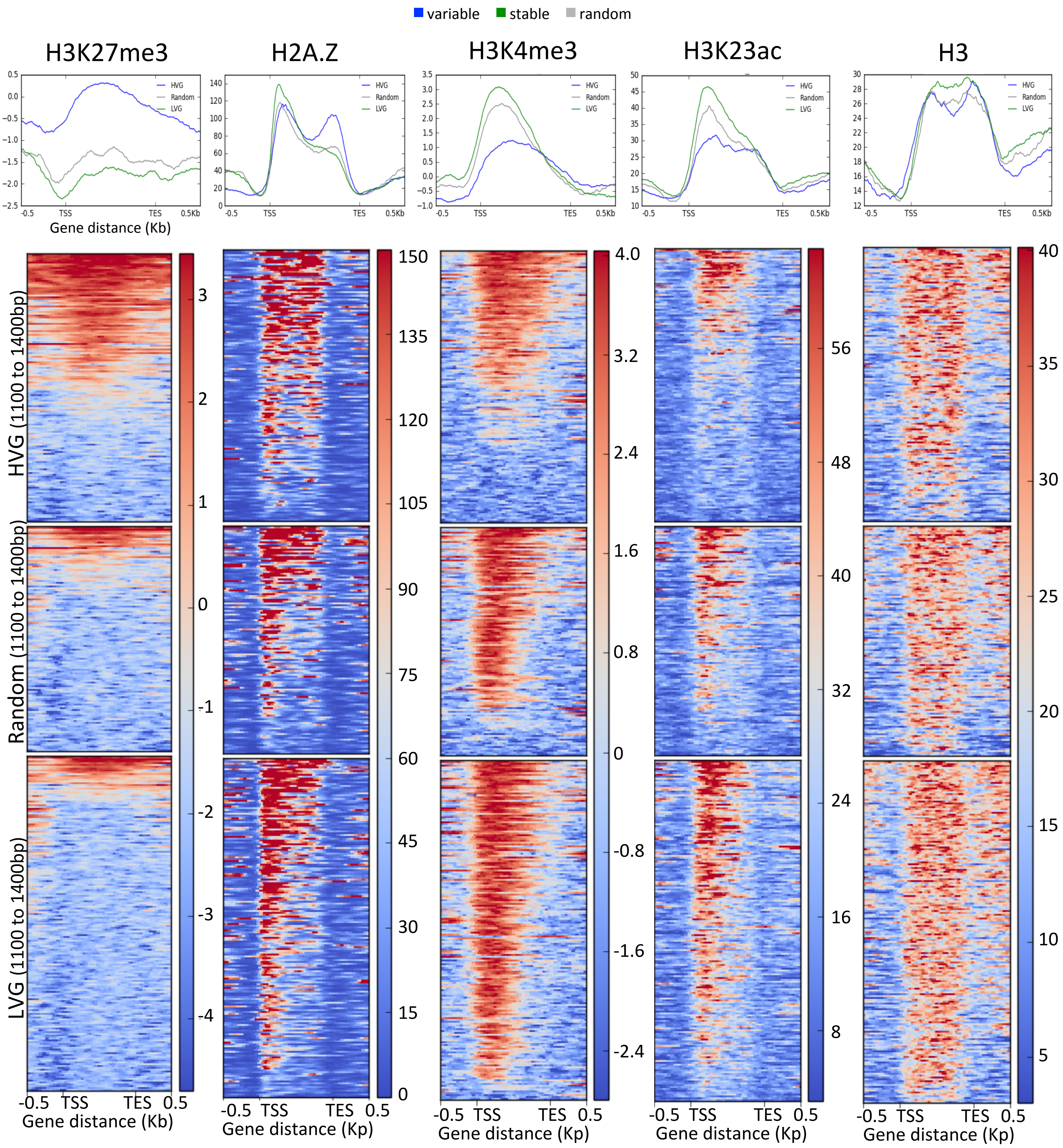

Appendix figure S7 (continues)

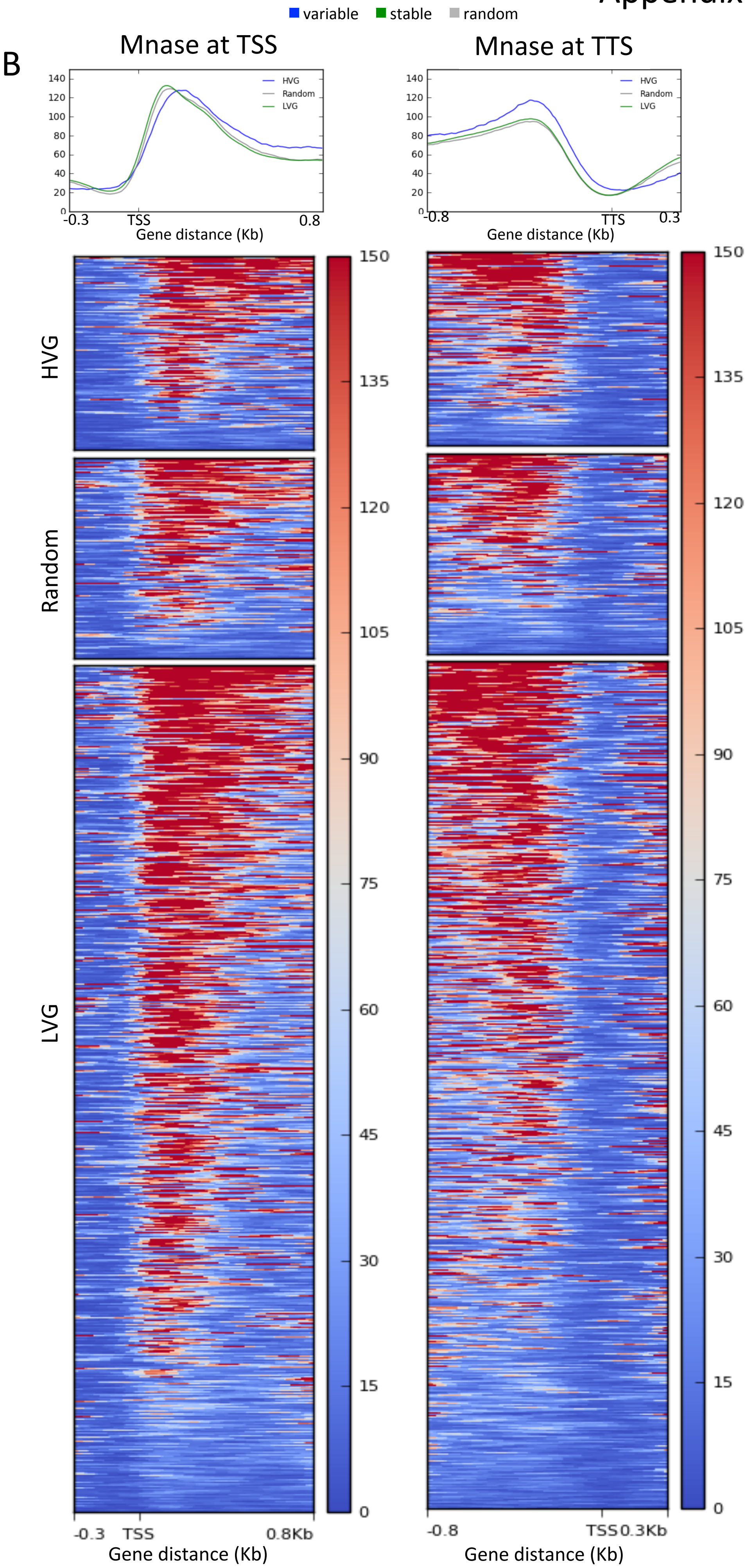

Appendix Figure S7:
A. Average profile and heatmap of H3K27me3, H2A.Z, H3K4me3, H3K23ac and H3 marks for HVGs (top), random genes (middle) and LVGs (bottom) that are 1100bp to 1400bp long. Red means a high level and blue means a low level for the chromatin marks.
B. Average profile and heatmap of MNase signal for HVGs (top), random genes (middle) and LVGs (bottom) around TSS (left) and TTS (right) of genes. Red means a high level and blue means a low level for the MNase signal.
